# Intravenous fluid restriction after major abdominal surgery: a randomized blinded clinical trial

**DOI:** 10.1186/1745-6215-10-50

**Published:** 2009-07-07

**Authors:** Hester Vermeulen, Jan Hofland, Dink A Legemate, Dirk T Ubbink

**Affiliations:** 1 Department of Quality Assurance & Process Innovation, Academic Medical Center, University of Amsterdam, Amsterdam, the Netherlands; 2 Department of Anesthesiology, Erasmus Medical Center, University of Rotterdam, Rotterdam, the Netherlands; 3 Department of Surgery, Academic Medical Center, University of Amsterdam, Amsterdam, the Netherlands

## Abstract

**Background:**

Intravenous (IV) fluid administration is an essential part of postoperative care. Some studies suggest that a restricted post-operative fluid regime reduces complications and postoperative hospital stay after surgery. We investigated the effects of postoperative fluid restriction in surgical patients undergoing major abdominal surgery.

**Methods:**

In a blinded randomized trial, 62 patients (ASA I-III) undergoing elective major abdominal surgical procedures in a university hospital were allocated either to a restricted (1.5 L/24 h) or a standard postoperative IV fluid regime (2.5 L/24 h). Primary endpoint was length of postoperative hospital stay (PHS). Secondary endpoints included postoperative complications and time to restore gastric functions.

**Results:**

After a 1-year inclusion period, an unplanned interim analysis was made because of many protocol violations due to patient deterioration. In the group with the restricted regime we found a significantly increased PHS (12.3 vs. 8.3 days; p = 0.049) and significantly more major complications: 12 in 30 (40%) vs. 5 in 32 (16%) patients (Absolute Risk Increase: 0.24 [95%CI: 0.03 to 0.46], i.e. a number needed to harm of 4 [95%CI: 2–33]). Therefore, the trial was stopped prematurely. Intention to treat analysis showed no differences in time to restore gastric functions between the groups.

**Conclusion:**

Restricted postoperative IV fluid management, as performed in this trial, in patients undergoing major abdominal surgery appears harmful as it is accompanied by an increased risk of major postoperative complications and a prolonged postoperative hospital stay.

**Trial registration:**

Current Controlled Trials ISRCTN16719551

## Background

Fluid administration during and after abdominal surgery is an essential part of postoperative care to maintain the patients' fluid and biochemical balance. Abdominal surgical procedures are associated with dehydration from preoperative fasting, bowel preparation, and intra- and post-operative fluid and electrolyte loss [1]. In clinical practice, in particular in major aortic and abdominal surgery, it is not uncommon to see very large amounts of fluids given, even in excess of actual losses [1]. Concern about preoperative fluid deficits, support of circulation and cardiac function after general and regional anesthesia, control of postoperative circulation, avoidance of blood transfusion and preservation of urine output are all issues that are thought to account for the administration of these excessive amounts of fluid [1]. However, in thoracic surgery a more "dry" regime is usually applied in order to prevent pulmonary complications [2]. That postoperative fluid overload might not be a benign problem has already been reported in 1990 [3].

At present, controversy exists as to the optimum volume of intravenous fluids during and after general surgery [4]. In minor (day-care) surgery for dental, laparoscopic or gynecological procedures, intraoperative fluid restriction (1–2 ml/kg) did not appear beneficial to patient recovery and well-being [5-9]. In larger surgical procedures, some advocated a high-volume intraoperative fluid intake (40 ml/kg) [10], or a postoperative intake on demand [11], while others described early oral liquid nutrient intake and discontinuation of intravenous fluid replacement to be beneficial [12]. Several small, randomized clinical trials suggested that a restricted intraoperative (4 ml/kg/h) or postoperative intravenous fluid regime (aiming at a constant body weight) may reduce hospital stay and postoperative complications [13], such as delayed gastrointestinal function and patient discomfort [14,15]. An explanation for this effect is thought to be a diminished ability of starved patients to excrete an excess of sodium and water postoperatively, which may cause a fluid overload that has adverse effects on gastrointestinal physiology and edema formation [1,16,17]. However, a recent report on patients undergoing elective colorectal surgery did not found a reduced hospital stay when a restriction of postoperative intravenous fluid and sodium was applied [18].

Thus, all the studies mentioned above showed ambiguous results. Furthermore, blinding was hardly ever used in these trials. This may have led to therapeutic interventions before the endpoint of the studies were reached, which in turn might have confounded the results.

Therefore, we designed and conducted a randomized, blinded, clinical trial in patients undergoing various major abdominal surgical interventions to compare the effect of a standard versus a restrictive postoperative intravenous fluid management on hospital stay, complication rate, and gastrointestinal function.

In our hospital the standard postoperative fluid regime is at least 2.5 litres/24 h of isotonic fluids. Before the start of this study, we performed an audit among all other university hospitals in the Netherlands on their standard fluid therapy after abdominal surgery. This audit confirmed that our postoperative fluid regime was in agreement with the current standard performed in the Netherlands [unpublished data]. Therefore, we chose 2.5 litres/24 h as amount for the standard postoperative intravenous fluid management and 1.5 litres/24 h for the restrictive fluid management in this study.

## Methods

### Patients

The Local Research Ethics Committee approved this study, and patients were included only after they had given their written informed consent. The trial was conducted according to the highest methodological standards to avoid bias and described according to the standards of the CONSORT statement. All consecutive adult patients with a physical status ASA I-III and scheduled for elective general abdominal surgery between May 2004 and July 2005 were eligible for inclusion. To obtain a representative sample of routine general major surgical procedures, all types of gastric resections, bowel procedures (small bowel, colon and/or rectum), bile duct restoring procedures, pancreaticoduodenectomies, or partial resections of the pancreas were included. Patients were excluded from the study if any of the following criteria were present: Scheduled for laparoscopic, liver or esophageal surgery and/or anticipated postoperative stay on the Intensive Care Unit, age <18 yrs, emergency operation, pregnancy, breastfeeding period, impaired renal function, significant cardiac disease (NYHA/CCS ≥ III), presence of diabetes mellitus, pre-operative IV drip-feeding, contraindications for applying epidural analgesia or failed attempt or logistical reasons.

### Procedures

Patients were approached and enrolled by one of the researchers (HV, DU or MSV) and subsequently randomly assigned to receive either a postoperative restricted intravenous (IV) fluid regime (RFR; 1.5 L/24 h) or a hospital standard IV fluid regime (SFR; 2.5 L/24 h). Statistical randomization was performed by means of a computer randomization program, to ensure allocation concealment. To balance both groups, minimization (a method of stratification) was performed for gender and age. The result of this computer randomization was enclosed in a sealed, opaque envelope and delivered to the nursing ward shortly before the operation. Also, the necessary equipment for the study (infusion pump, IV-line, clothing bag to blind the infusion system, and the relevant case report forms) were delivered. The sealed envelope, together with all these materials, was sent with the patient entering the operation room. Disclosure of the randomization took place at the end of the operation by opening the sealed envelope.

### Clinical management

All patients were admitted the day before surgery. Preoperative bowel preparation regime (two enemas), fasting regime, pre-operative medication, and postoperative nasogastric intubation were according to the ruling standards [19]).

Just before surgery a routine epidural catheter was placed in all patients. Anesthesia for all surgical procedures consisted of a combination of epidural analgesia together with a balanced anesthesia technique according to the attending anesthetist. Only the intraoperative IV fluid infusion regime was according to a standardized protocol in order to avoid great variation in fluid management just before the real study randomization took place; i.e. opening the sealed envelope at the end of the operation (Appendix). One of the researchers (JH or MD) supervised adherence to this protocol and they also disconnected the IV lines after the surgical procedure. At the end of the surgical procedure, as described above, the randomization envelope was opened. Then, only one IV catheter was re-connected to a new IV fluid line that was led through an infusion pump (Infusomat^® ^P; B. Braun Medical Inc., Bethlehem, PA, USA) and was connected to five (SFR; 2.5 L/24 h) or three (RFR; 1.5 L/24 h) 500 ml fluid bags of Ringer's Lactate solution simultaneously to secure a constant fluid administration for the first 24 hrs after surgery, starting upon arrival at the recovery room. The pump infusion rate was set according to the allocated IV fluid regime. Subsequently, SFR patients received 1500 ml 0.9% NaCl and 1000 ml 5% glucose, while RFR patients received 1000 ml 0.9% NaCl and 500 ml 5% glucose IV. Patients, attending physicians, and nurses on the wards were all blinded to the treatment given. This was ensured by immediate covering of the infusion bags and pump by means of an opaque clothing bag. To maintain this blinding on the wards, an independent nurse who was not assigned to care for the patient was charged to change the infusion bags every 24 hrs and/or solve any pump problems. A sheet with pre-defined criteria for discontinuation of the blinding (and to change the fluid regime if necessary) was attached to the clothing bag. Hence, as long as these criteria were not met, the fluid regime was continued according to the initial allocation.

Criteria for discontinuation of the blinding were: mean arterial blood pressure (MAP) below 50 mm Hg, acute heart failure, indication for re-operation, and signs of severe blood loss or shock. Although the presence of urine flow indicates blood flow to the kidney, oliguria was not considered a reliable sign of pending renal dysfunction [20,21].

In both groups, postoperatively, the nasogastric tube was removed directly after surgery or on the first postoperative day. Subsequently, patients were free in their oral fluid intake and received the allocated IV fluid regime until the attending physician judged this fluid administration could be discontinued, based on evaluation of the oral intake and bowel movements of the patient. We choose routine clinical practice as much as possible to proceed in this phase and not to use predefined criteria in order to let this study run as near to common clinical practice as reasonably possible. This chosen strategy is recently supported by the presented guidelines by Lassen too [22].

Until discontinuation of the IV fluid administration, it was impossible to use the daily fluid balance as a clinical monitoring variable because of the blinding. Instead, the attending residents monitored the patients on the basis of other parameters, such as the patients' clinical condition, wound appearance, oral fluid intake, peristalsis, urinary output, heart rate, blood pressure, and routine laboratory test results (e.g. hemoglobin, hematocrit, electrolytes, infection parameters, and kidney function). In the postoperative period, it was not possible to obtain patients' weights on a daily basis.

Postoperative analgesia consisted of administering bupivacaine (0.125%) mixed with fentanyl (2.5 mcg/ml) via the epidural catheter into the epidural space, together with paracetamol given orally or rectally (1 g, 4 times a day). After discontinuation of the epidural infusion the resident managed the further postoperative (analgesic) treatment and eventual dismissal according to common clinical practice rules.

### Endpoints

Primary endpoint was length of postoperative hospital stay (PHS), counted from the date of operation, as this parameter is commonly used in similar studies. Discharge criteria were: restored peristalsis (i.e. flatus, or defecation less than 8 times a day), unhampered oral intake of food and drink, and sufficient mobility to wash and dress. If a patient had received a stoma, its output should be less than 1 l/day. Secondary endpoints were major and minor postoperative complication rates, time to first passage of flatus and feces, discontinuation of IV fluids and return to normal diet (all intervals counted from the operation date). Postoperative complications were recorded according to the National Surgical Adverse Event Registration from the Dutch Society for Surgery during the in-patient period. The following complications were defined as 'major': death, cardiac events (i.e. myocardial infarction, arrhythmias, or admission to Coronary Care Unit), anastomotic leakage (based on CT-scan or findings during re-operation), sepsis, kidney failure requiring dialysis, and re-admission (i.e. disease-related re-hospitalization within 30 days after discharge; in these cases the reason for re-admission was not counted as additional complication). Relatively 'minor' complications were: abdominal wound abscess, infection or dehiscence, not requiring surgical reintervention, respiratory disorders or infection, bleeding, peripheral thrombo-embolism. Kidney function was not defined as endpoint because in the study by Lobo et al. none of the patients in the restricted group became oliguric or had a blood urea concentration above the upper limit of normal [17].

### Sample size calculation

To detect a reduction in PHS of 3 days (SD 5 days), which is in agreement with the study of Lobo et al. [17], with a two-sided 5% significance level and a power of 80%, a sample size of 50 patients per group was necessary, given an anticipated dropout rate of 10%. To recruit this number of patients a 12-month inclusion period was anticipated.

### Data collection

Data on patient characteristics, complications, and restoration of gastric function were extracted from the (electronic) patient dossiers and case report forms (HV, MS). Raw data regarding the primary and secondary endpoints were checked by an independent investigator (GB) and all disagreements were referred to a third reviewer (DU).

### Data safety monitoring board

To ensure a proper execution of the trial and to monitor the progress, outcome, and patient safety during the trial, its progress and any occurring adverse events were discussed regularly with an expert team (JH, DL, MD) that was not involved in the randomization procedure or postoperative treatment of the patients. No stopping rules were defined beforehand.

### Statistical analysis

Continuous data are presented as means and standard deviations (SD) or medians and interquartile ranges (IQR) if they were unevenly distributed. Differences were tested statistically using the Mann Whitney U-test. A p value < 0.05 was considered significant. For the analysis of categorical data we calculated the Absolute Risk Reduction (ARR) with a 95% confidence interval (95%CI). Data were analyzed using an intention to treat and per protocol principle. To detect possible factors associated with the occurrence of major postoperative complications, we performed stepwise multivariable logistic regression analysis.

For these analyses, SPSS for Windows version 12.0 (SPSS Inc., Chicago, Ill, USA) software was used. We did not schedule an interim analysis.

## Results

**Figure 1:**
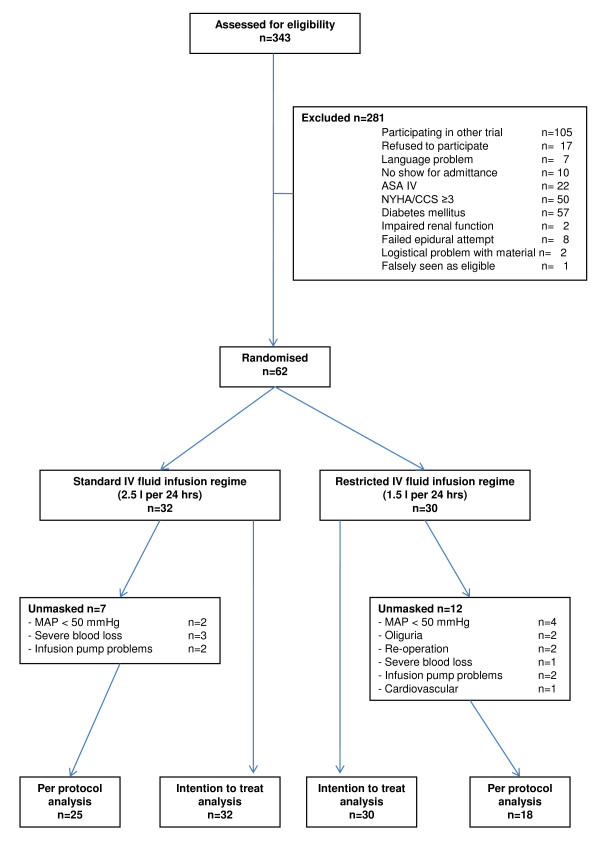
**Flow chart of patient inclusion, follow-up, and analysis**.

During the inclusion period 343 patients were potentially suitable for the study. After screening, 271 patients did not match all inclusion criteria and/or had one or more of the exclusion criteria. The flow chart of the number of patients at inclusion, during follow-up and available for analysis is shown in figure 1. At the end of the anticipated one-year inclusion period, the desired number of 100 patients was not reached, which jeopardized continuation of the trial as we had formulated this timeframe beforehand in our study protocol. Analyzing reasons for not reaching the expected inclusion level showed that roughly one third of eligible patients participated in other clinical trials. It was expected this problem would maintain when continuation/extension of the trial should be decided. Furthermore, protocol violations were observed rather frequently because of postoperative hypotension (remaining above a mean of 50 mmHg, but resulting in the administration of an extra bolus of saline i.v.), oliguria (also resulting in the administration of extra saline i.v.) or infusion pump problems (leading to a lower amount of fluid administered). This also was thought to be a significant problem for continuation of the study, however desired. Finally, nurses and residents, although unaware of the amount of fluid given, suspected none of the trial patients received the anticipated benefit from a restricted fluid regime. All these items were discussed among the investigators and the members of the safety monitoring board. Then, the decision was made to stop inclusion and to perform an unplanned interim analysis of the available data.

We therefore analyzed the results of 62 patients that were included after one year and had entered the protocol. Table 1 shows the characteristics of these patients. Duration and type of surgical procedures were similar in both treatment arms. Most patients underwent major surgery of the biliary or pancreatic region.

**Table 1:** Baseline patient characteristics.

	**Restricted group** **(n = 30)**	**Standard group** **(n = 32)**	p-value*
**Characteristics**			
Age (yrs)	55.5 (15.4)	53.6 (15.0)	0.623
Sex (Male/Female)	19/11	21/11	0.853
Height (m)	1.73 (0.08)	1.76 (0.08)	0.810
Weight (kg)	69.9 (12.5)	76.5 (17.1)	0.908
BMI	23.2 (4.2)	24.5 (4.7)	0.408
ASA			0.908
1	4 (13%)	5 (16%)	
2	21 (70%)	24 (75%)	
3	5 (17%)	3 (9%)	
Type of surgery			0.522
-Gastric	0 (0%)	1 (3%)	
-Pancreas	14 (47%)	11 (34%)	
-Bile duct	7 (23%)	9 (28%)	
-Gall bladder	0 (0%)	1 (3%)	
-Small bowel	2 (7%)	3 (9%)	
-Colon	3 (10%)	4 (13%)	
-Rectum	3 (10%)	1 (3%)	
-Adrenal gland	0 (0%)	1 (3%)	
-Retroperitoneal tumor	0 (0%)	1 (3%)	
-Explorative laparotomy	1 (3%)	0 (0%)	

Values are means (± SD) or numbers (%)

BMI = Body Mass Index

*: Student t-test for comparison of means; Kruskall-Wallis test for categorical variables

Although the intra-operative IV fluid management actually given during surgery was higher than the prescribed regime, no statistical significant differences were found between the groups (Table 2). Also, the intra-operative amounts of blood-loss, blood product administration, surgical procedure time, urine production and post-operative length of given epidural analgesia were not different between the groups (Table 2).

**Table 2:** Perioperative parameters

	**Restricted group** **(n = 30)**	**Standard group** **(n = 32)**	**P-value**
Insertion level of epidural catheter			
-Mid-thoracic (T 6–9)	22 (73%)	25 (78%)	0.77
-Low-thoracic (T 10–12)	3 (10%)	3 (9%)	1.0
-Lumbar	5 (17%)	4 (13%)	0.73
Time of epidural analgesia (days)	3.3 (1.5)	3.7 (1.5)	0.41
Duration of surgical procedure (hrs)	4.3 (2.1)	4.2 (1.7)	0.83
Blood loss during procedure (ml)	450 (435 to 1089)	500 (424 to 1688)	0.65
Urine production during procedure (ml)	315 (249 to 531)	290 (248 to 529)	0.86
IV crystalloids (Ringer's Lactate)			
1^st ^hr (ml.kg^-1 ^bodyweight)	20 (6.3)	19 (5.5)	0.45
2^nd ^etc. hrs (ml.kg^-1 ^bodyweight)	8.3 (4.1)	9.0 (3.7)	0.52
IV colloids (HAES-Steril^® ^6%) (l)	0.9 (0.5)	1.1 (0.9)	0.13
Patients requiring PRC	21%	11%	0.35

PRC = Packed Red Cells. Two patients in the restricted group and one in the standard group required 2 PRC units or more.

Values are expressed as means (± SD) or numbers (%). For blood loss and urine production, medians with 95% Confidence Intervals are given.

In 19 patients unmasking and discontinuation of the study protocol occurred (SFR vs. RFR: 7/32 vs. 12/30; Fisher's exact test: p = 0.17). Reasons for unmasking are shown in figure 1. Thus, 43 patients could be included in a per protocol analysis.

In an intention to treat analysis, PHS was significantly longer in the RFR group; 12.3 vs. 8.3 days; p = 0.049 (Table 3). This difference was not found in the per protocol analysis (Table 4). No significant differences were found between both groups for any of the gastrointestinal function parameters, times to removal of nasogastric tubes, and discontinuation of IV and epidural catheters (Tables 3 and 4). None of the patients suffered from postoperative acute kidney injury.

**Table 3:** Intention to treat analysis of the primary and secondary endpoints

	**Restricted** **(n = 30)**	**Standard** **(n = 32)**	**Absolute Risk Reduction (95% confidence interval)**
Postoperative hospital stay (days), mean (SD)	12.3 (12.7)	8.3 (4.5)	
Median (IQR)	9.0 (6.8–11.3)	7.0 (6.0–9.8)	0.049*
**SECONDARY ENDPOINTS**			
***Postoperative complications***	23	13	0.360 (0.133 to 0.588)
- **Major complications**	12	5	0.244 (0.028 to 0.460)
- Death	1	0	
- Cardiac	2	0	
- Leakage of anastomosis	6	1	0.135 (-0.011 to 0.282)
- Re-admission	3	4	
- **Minor complications**	11	8	0.117 (-0.112 to 0.345)
- Wound infection	5	1	
- Wound dehiscence	1	0	
- Infection	0	1	
- Respiratory	1	0	
- Bleeding	1	3	
- Thromboembolism	1	0	
- Miscellaneous	2	3	
**Gastric function**			
- First flatus (days)	2.8 (1.3)	2.9 (1.5)	
Median (IQR)	3 (2–3)	3 (1–4)	0.713*
- First defecation (days)	3.7 (1.3)	3.5 (1.7)	
Median (IQR)	4 (3–4)	4 (2–5)	0.725*
- Removal nasogastric tube (days)	2.3 (1.3)	2.4 (2.9)	
Median (IQR)	2 (1–3)	2 (1–2)	0.393*
- Removal of epidural catheter (days)	3.3 (1.5)	3.7 (1.5)	
Median (IQR)	4 (2–4)	4 (3–4)	0.420*
- Removal of IV catheter (days)	4.9 (2.7)	5.1 (4.5)	
Median (IQR)	4 (3–5)	4 (3–5)	0.648*
- Normal diet (days)	4.3 (1.9)	4.2 (3.2)	
Median (IQR)	4 (3–5)	4 (3–4)	0.254*

* = p-values according to Mann-Whitney U test

**Table 4:** Per protocol analysis of primary and secondary endpoints

	**Restricted** **(n = 18)**	**Standard** **(n = 25)**	**Absolute Risk Reduction (95% confidence interval)**
**Primary endpoint**			
Postoperative hospital stay (days), mean (SD)	7.9 (2.4)	7.3 (3.0)	
Median (IQR)	7.0 (6.0–10.0)	7.0 (5.5–8.0)	0.337*
**Secondary endpoints**			
***Postoperative complications***	4	6	-0.018 (-0.273 to 0.237)
- **Major complications**	1	3	-0.064 (-0.230 to 0.101)
- Re-admission	1	3	
- **Minor complications**	3	0	0.047 (-0.168 to 0.261)
- Wound infection	3	0	
Gastric function			
- First flatus (days)	2.5 (1.2)	2.8 (1.5)	
Median (IQR)	3 (1.3–3)	3 (1–4)	0.569*
- First defecation (days)	3.5 (1.2)	3.3 (1.6)	
Median (IQR)	4 (2.8–4)	4 (2–5)	0.958*
- Removal nasogastric tube (days)	2.1 (1.2)	1.8 (1.2)	
Median (IQR)	2 (1–3)	1(1–2)	0.471*
- Removal of epidural catheter (days)	3.5 (1.3)	3.6 (1.3)	
Median (IQR)	4 (3–4)	4 (3–4)	0.707*
- Removal of IV catheter (days)	3.9 (1.1)	3.8 (1.3)	
Median (IQR)	4 (3–5)	4 (3–5)	0.801*
- Normal diet (days)	3.8 (1.6)	3.6 (1.7)	
Median (IQR)	4 (2–5)	3 (3–4)	0.577*

* = p-values according to Mann-Whitney U test

Significantly more (major) postoperative complications (especially anastomotic leakage) were found in the RFR group (Table 3), with a Number Needed to Harm of 4 (95% CI: 2–36). This difference disappeared in the per protocol analysis (Table 4). Anastomotic leakage occurred in patients who underwent colon surgery (n = 4), pancreaticojejunostomy (n = 2), or hepaticojejunostomy (n = 1). No significant differences were found between the groups for the minor complications or restoration of gastric functions. In order to avoid the risk of false-positive findings in our small sample size we refrained from subgroup analysis. Multivariable logistic regression analysis of potential factors contributing to the occurrence of complications did not indicate any significant confounders.

## Discussion

This randomized clinical trial shows that a restricted postoperative intravenous fluid management in rather complex patients undergoing major abdominal surgical procedures under a combination of epidural and balanced general anesthesia can induce an increased risk of developing (major) postoperative complications together with a prolonged postoperative hospital stay, and therefore can be harmful for such patients. Based on previous reports, a reduction of postoperative fluid supply was expected to be beneficial in terms of postoperative complications and hospital stay. Therefore, we were surprised by the contrasting findings of this trial regarding these very endpoints.

The trial was terminated after one year of inclusion, although the preset number of patients enrolled into the study was not met, e.g. because the clinical situation of several included patients deteriorated postoperatively to such an extent that the surgeon in charge was quite uncomfortable with the (unknown) amount of fluid given and decided to unmask the treatment, thereby causing several protocol violations. No significant difference between the number of patients unmasked in the SFR group vs. the RFR group was found. It is reasonable to infer that patients after unmasking will have received extra fluid, although the exact amount was not recorded in detail. However, it is highly unlikely that thereby the RFR group could have received more fluid in total than the SFR group. Furthermore, it is known that protocol violations in a trial can best be handled by performing an intention-to-treat analysis because any other analysis might introduce bias [23]. So, our findings are in contrast to previous reported trials that suggest postoperative fluid restriction to be beneficial and safe [13,14,18]. Furthermore, the methodological quality of this trial is high as compared to previous trials. This is based on the allocation concealment, the blinding of patients, treating surgeons and outcome assessors, and the data acquisition and checking by two investigators independently.

Definitions of a 'liberal' fluid regime as used by different authors are quite variable [10,19]. This also holds for fluid 'restriction', which ranges from maintaining preoperative body weight to 2 l in the first 24 h [14,18]. The different effects found may be explained by several reasons. Firstly, the patients included in this study underwent a wider range of more major surgical interventions under epidural analgesia. This was neither the case in the study of Lobo et al. [17], in which all patients underwent hemicolectomies or sigmoidectomies, nor in the studies by Brandstrup et al. [14] and MacKay et al. [18], who included only patients undergoing colorectal resections, one third of whom performed via laparoscopy. Secondly, in the study of Brandstrup et al., considerably more patients with ASA 1 classification (almost 50%) were included [14]. This is in contrast with our study, where most patients were classified as ASA 2 and 3(about 85%). Lobo et al. did not report the ASA classification, but their exclusion criteria very likely prohibited inclusion of patients classified as ASA 3 [17]. Thus, our patients were at a higher perioperative risk due to the higher prevalence of co-morbidity and therefore, they may have benefited from a more conventional fluid intake. Thirdly, in our study an extensive blinding procedure was followed of patients, physicians, as well as nurses, whereas in the studies by Lobo et al. [17], Brandstrup et al. [14], and MacKay et al. [18], only the outcome assessor was blinded. This single blinding may have caused performance bias in favor of the restricted fluid regime in those studies. On the other hand, a small study by Holte et al. did not show a clear benefit from fluid restriction in fast track surgical patients. Rather, they found a tendency to increased morbidity in the fluid restricted group [21].

The administration of IV fluid to avoid dehydration and maintenance of circulating volume with prevention of inadequate tissue perfusion should be considered along with maintenance of hypnosis, pain relief and muscle relaxation, a cornerstone of anesthesia practice [24]. The effect of anesthetic and postoperative analgesic techniques on outcome varies with the type of operation that is performed [25]. Although, most adverse morbid outcomes in high-risk patients undergoing major abdominal surgery are not reduced by use of a combined epidural and general anesthesia technique, the improvement in analgesia and reduction in respiratory failure and the low risk of serious adverse events makes it likely that high-risk patients undergoing major intra-abdominal surgery will benefit from this combination technique [26,27]. Nevertheless, after the publication of the MASTER study, the use of this combination of techniques has declined in some parts of the world [28].

Compensatory intravascular volume expansion, necessary to compensate for venous dilation and cardiac depression due to anesthesia and external and third space losses, is the classic argument used for fluid administration guidelines [29]. All our patients received an epidural and during operation a combination of epidural analgesia together with balanced anesthesia was used in all of them. To compensate for cardiovascular effects, both patient groups received 500 ml hydroxyethyl starch at the start of the operation procedure (Appendix). By standardizing the intra-operative IV fluid regime both patient groups reached the point of randomization, just after surgery was finished, in a more or less equal "filling state" also; i.e. no significant differences were found in the perioperative variables shown at Table 2. Therefore it seems unlikely that the RFR group could have had much added harm of the restricted fluid regime in comparison with the standard fluid regime by having epidural analgesia.

Recently published reviews address the struggle of perioperative fluid management decision making in clinical practice due to the lack of evidence for choosing a "wet" vs. "dry" regimen for a particular surgical procedure [1,4,24]. These studies not only suggested to study more patients undergoing specific surgical procedures, but also to examine intermediate fluid regimens [1,4,24]. Another difficulty with properly interpreting the results of the published studies is the difference in time schedules regarding the applied fluid infusion regime. In the study of Lobo and colleagues the patients in the standard group received a water load of 40 ml/kg (around 20 ml/kg/h) and in the restricted group of 34 ml/kg (around 17 ml/kg/h) during the operation [17]. However, they merely randomized the post-operative fluid management 3 L (1 L saline and 2 L dextrose 5%) vs. 2 L (0.5 L saline and 1.5 L dextrose 5%) [17]. The study of Brandstrup and colleagues not only randomized the complete perioperative fluid infusion regimens, but also mixed colloid and crystalloid regimens, which makes the study very difficult to interpret [14]. Nisanevich and colleagues randomized the intraoperative fluid regime (around 15 ml/kg/h vs. 4 ml/kg/h) and blinded the postoperative caregivers, which resulted in administering around 2 L fluid/day postoperatively in both groups of patients, of which 65–70% had underwent colorectal surgery [15]. In our study, in which around 66% of the patients underwent pancreatic or bile duct surgery, the applied intraoperative fluid management can be considered as an intermediate fluid regime (around 11 ml/kg/h). Therefore, the differences found can be explained by the variation in postoperative fluid regime only.

Many clinicians would prefer to titrate fluid therapy to some form of clinical response. Historically, e.g. urine output was considered such a monitoring tool. Lobo et al however, found no correlation between the allocated fluid regime and urine output; i.e. none of their patients became oliguric or showed a concentration increase of urea in blood above the upper limit of normal and the restricted group had a decreased PHS [17]. The results of our study just showed the opposite for PHS. Given this evidence and the fact that PHS may be influenced by many other factors besides the impossibility to titrate fluids in this trial, the artificial construct of having postoperative fluid therapy restricted to a fixed rate cannot be the only cause for the result. Still, tissue perfusion indices as goals for fluid management stay intuitively very appealing [4]. Although at present e.g. Stroke Volume Variation and Esophagus Doppler are subject of research, use of this information to guide fluid therapy must still gain support from clinical trial data [4,30]. The mentioned monitors can e.g. easily be used in an ICU setting, but are less or not suitable for monitoring purposes in patient admitted at a regular ward and therefore will have less efficiency for influencing longer term outcomes as PHS.

Because we performed an outcome study instead of a pathophysiological study, in which measurements of different fluid compartments usually are subject of study, it is difficult to provide a pathophysiological explanation of the increased complication rate in the RFR group. Inflammation, effects on microcirculation, induced cellular leakage, differences in fluid load are amongst items that may play a role [31]. Moreover, recently it became clear that the theoretical models used to describe mechanisms of water movements, e.g. in the digestive tract are incomplete [32].

Our study showed an unexpected high anastomotic leakage rate (6 out of 30 patients) in the restricted regime group, for which we do not have an explanation, but which is in contrast with the study of Brandstrup et al. [14]. Apart from the existing belief in the conventional fluid administration and the cumbersome execution of the trial, the observed harmful effects led to the decision to prematurely stop the trial. However, the clearly significant findings cannot only be explained by a possibly false-positive result, as might be the case in trials stopped for early benefit [33]. Our per-protocol analysis did not show any significant differences between groups, which is not surprising as in this analysis the patients with complications were not included.

### Lessons learnt from this trial

We designed a trial in which all stakeholders were blinded to the treatment given in order to tackle many possible sources of bias and, at the same time, to stay as close as possible to common clinical practice. In trials about fluid regimens this blinding procedure appears feasible. However, in this trial the many patients with hypovolaemia and low blood pressure led to a substantial number of protocol violations. A weight-based regime together with stricter predefined daily laboratory testing may enable better protocol adherence. On the other hand, additional measures to assess efficacy in a trial, e.g. directly postoperative body weight measurements, lab testing, or even more attention to the patient's condition, are beyond common clinical practice. This will weaken the implications for real life that can be drawn from such a study. Furthermore, the inclusion speed in this study was lower than expected, because many eligible patients could not be included as they were already participating in other concurrent clinical trials. Future trials should try and include a consecutive set of eligible patients or be conducted in a multicenter fashion in order to obtain sufficient and generalisable data. Finally, our conclusion contrasts with the results of other studies. This might be the result of the rigid design including the blinding procedure and the fact that the large majority of patients underwent major surgery. Hence, results from trials in this multifaceted area of research should be interpreted carefully and with an eye on the case-mix of patients included.

In conclusion: this trial suggests that a restricted postoperative IV fluid regime of 1.5 L/24 h appears harmful to patients undergoing major abdominal surgery. The findings should be reconfirmed in a larger trial of good design and conduct. In such a trial one should anticipate the threat of protocol violation as a result of (elaborate) blinding.

## Abbreviations

ARR: Absolute Risk Reduction; ASA: American Standards Association; BMI: Body Mass Index; CCS: Canadian Cardiovascular Society; CI: Confidence Interval; HAES: Hydroxyaethyl Starch; Hb: Hemoglobin; IQR: Inter Quartile Range; IV: IntraVenous(ly); MAP: Mean Arterial blood Pressure; NYHA: New York Heart Association; PHS: Postoperative Hospital Stay; PRC: Packed Red Cells; RFR: Restricted Fluid Regime; SD: Standard Deviation; SFR: Standard Fluid Regime; SPSS: Statistical Package for the Social Sciences.

## Competing interests

The authors declare that they have no competing interests.

## Authors' contributions

HV performed patient inclusion and data analysis, and drafted the manuscript. JH contributed to the execution of the trial, data interpretation, and writing and critical review of the manuscript. DAL participated in the trial design, interpretation of the data, and critical review of the manuscript. DTU conducted patient inclusion, data analysis and interpretation, writing and critical review of the manuscript. All authors read and approved the final manuscript.

## Appendix

### Standardized intra-operative IV fluid regime

Basic IV infusion regime by giving crystalloids during the surgical procedure:

-1^st ^hour of the procedure: 20 ml per kg body weight of Ringer's Lactate

-2^nd ^and further hours: 6 ml per kg body weight of Ringer's Lactate

Colloid IV infusion and blood transfusion regime to compensate for cardiovascular effects of epidural analgesia and blood loss during the surgical procedure:

-500 ml hydroxyethyl starch (HAES-Steril^® ^6% [Fresenius-Kabi AG, Bad Homburg v.d. H., Germany]) was given at the start of the procedure

-When blood loss ≥ 500 ml, a 2^nd ^500 ml hydroxyethyl starch (HAES-Steril^® ^6%) was given

-When blood loss ≥ 1,000 ml, a 3^rd ^500 ml hydroxyethyl starch (HAES-Steril^® ^6%) was given

-When blood loss ≥ 1,500 ml:

• Packed Red Cells (PRC) were given guided by Haemoglobin (Hb) level

Patient < 70 yrs: trigger Hb-level = 5·0 mmol.l^-1 ^(= 8·1 g.dl^-1^)

Patient ≥ 70 year: trigger Hb-level = 5·5 mmol.l^-1 ^(= 8·9 g.dl^-1^)

• When > 2 units PRC were necessary: 2 units PRC were alternated with 1 unit plasma in order to maintain adequate blood coagulation

• If the trigger Hb-level was not met it was allowed to give a 4^th ^hydroxyethyl starch (HAES-steril^® ^6%) but only in case the first one was being administered ≥ 6 h ago. Otherwise IV infusion of Ringer's lactate was then given
